# Comparison Between the In-Hospital Outcomes of Patients Presented With Acute Anterior Wall ST-Segment Elevation Myocardial Infarction With and Without a Right Bundle Branch Block

**DOI:** 10.7759/cureus.80385

**Published:** 2025-03-11

**Authors:** Muhammad Zohaib Amjad, Bushra Mumtaz, Muhammad Abubakar, Muhammad Irfan Jamil, Muhammad Awais, Adnan Ahmad Zafar, Adeel Ahmed, Hibba Tul Noor

**Affiliations:** 1 Internal Medicine, Royal Berkshire Hospital, Reading, GBR; 2 Cardiology, Quaid-E-Azam Medical College, Bahawalpur, PAK; 3 Cardiology, DHQ Hospital Mandi Bahauddin, Lahore, PAK; 4 Cardiology, Multan Institute of Cardiology, Multan, PAK; 5 Nephrology, Lahore General Hospital, Lahore, PAK; 6 Medicine, Lahore General Hospital, Lahore, PAK; 7 Nephrology, Islam Medical College, Sialkot, PAK; 8 Cardiology, Punjab Institute of Cardiology, Lahore, PAK

**Keywords:** in-hospital outcome, mortality, post-myocardial infarction, right bundle branch block, st-elevation myocardial infarction (stemi)

## Abstract

Background: Although a right bundle branch block (RBBB) complicates anterior wall ST-segment elevation myocardial infarction (AW-STEMI), its independent prognostic significance remains understudied.

Material and method: This cross-sectional observational study was conducted at the Punjab Institute of Cardiology, Lahore, over a period of 18 months from January 2022 to June 2023. A total of 349 patients presenting with acute AW-STEMI were enrolled. They were stratified into RBBB and non-RBBB groups. Outcomes included mortality, cardiogenic shock, cardiac arrest, arrhythmias, complete heart block (CHB), post-myocardial infarction (MI) angina, and hospital stay duration. The effect of confounding variables on in-hospital mortality was evaluated through stratification among the two groups.

Results: AW-STEMI with RBBB was reported in 50 (14.3%) out of 349 patients. Both groups had a similar mean age (p = 0.276), and comorbidities, including hypertension (p = 0.363), diabetes mellitus (p = 0.872), chronic kidney disease (CKD) (p = 0.299), dyslipidemia (p = 0.486), smoking status, and prior myocardial infarction (p > 0.05), were comparable. Left ventricular ejection fraction (LVEF) was significantly lower in RBBB patients (p < 0.001). Peak troponin-I levels were significantly higher in the RBBB group (p < 0.001, 95% confidence interval (CI): 6.581-11.803). In-hospital mortality was significantly higher in RBBB patients (16% vs. 5.7%, p = 0.009, odds ratio (OR) = 3.160, 95% CI: 1.284-7.777). Cardiogenic shock occurred more frequently in RBBB patients (36% vs. 16.4%, p = 0.003, OR = 2.672, 95% CI: 1.394-5.120). Arrhythmias were significantly higher in the RBBB group (42% vs. 19.7%, p = 0.001, OR = 2.946, 95% CI: 1.569-5.529). Cardiac arrest (16% vs. 11.4%, p = 0.354) and post-MI angina (24% vs. 15.4%, p = 0.170) were more common in RBBB patients but were not statistically significant. CHB was observed in 12% of RBBB patients vs. 8% in non-RBBB (p = 0.345). CKD was strongly associated with increased mortality, with all affected STEMI patients with RBBB experiencing fatal outcomes, whereas those without RBBB had significantly lower mortality. The choice of reperfusion strategy played a crucial role, with primary PCI demonstrating a survival benefit in RBBB patients, while thrombolysis and medical management were linked to markedly higher mortality rates in this group.

Conclusion: Patients with AW-STEMI and RBBB had significantly worse in-hospital outcomes, including higher mortality, increased risk of cardiogenic shock, and a greater prevalence of arrhythmias. Although cardiac arrest, post-MI angina, and CHB were more frequent in RBBB patients, these differences were not statistically significant. Primary PCI was associated with a lower mortality risk in RBBB patients.

## Introduction

Acute coronary syndrome (ACS), particularly ST-segment elevation myocardial infarction (STEMI), remains a major health concern, affecting millions globally [1]. Recent data estimate that approximately 7.1 million individuals worldwide are diagnosed with ACS annually, with STEMI accounting for nearly 30% of cases. In the United States, the incidence of myocardial infarction (MI) is estimated at 805,000 cases per year, comprising 605,000 new cases and 200,000 recurrent events, based on the latest 2023 statistics [2,3]. Conduction abnormalities, such as a right bundle branch block (RBBB), worsen the clinical outcomes for these patients. Historically, RBBB has been perceived as less ominous than LBBB. LBBB is classically linked to proximal left anterior descending (LAD) artery occlusion and severe left ventricular dysfunction, leading to a well-established correlation with increased mortality and adverse cardiac events. However, recent studies challenge this notion, demonstrating that RBBB in STEMI is an independent predictor of poor prognosis, including increased mortality, cardiogenic shock, and conduction disturbances [4]. Despite representing a smaller fraction of ACS presentations, the presence of RBBB has been associated independently associated with a 66% increased risk of 30-day in-hospital mortality and higher rates of acute heart failure (hazard ratio (HR) 1.37), complete heart block (CHB) (HR 2.90), and permanent pacemaker implantation (HR 2.51) [5,6]. Hashim et al. found the mortality rate to be 23.3%, emphasizing the deadliness of STEMI with context RBBB [7].

The pathophysiology underlying RBBB’s adverse impact may involve extensive myocardial necrosis impairing the right bundle’s conduction pathway, often indicative of larger infarct size or proximal LAD artery occlusion. Furthermore, RBBB may delay the diagnosis of STEMI due to atypical electrocardiographic patterns, potentially postponing revascularization, a critical determinant of outcomes [8,9].

Despite the growing recognition of RBBB’s prognostic value, several knowledge gaps persist. First, existing studies predominantly focus on mortality and selected complications with limited data on cardiac arrest, post-MI angina, arrhythmias, and cardiogenic shock. Second, the influence of reperfusion strategies (e.g., primary PCI vs. medical management) on RBBB-associated outcomes, including duration of hospital stay and frequency of CHB, remains underexplored. Third, most previous studies have been retrospective in nature. However, this study will prospectively collect data to reduce bias, improve data accuracy, and ensure real-time outcome assessment. This study addresses these gaps by evaluating a comprehensive set of in-hospital outcomes to clarify the clinical burden of RBBB in AW-STEMI and optimize evidence-based management.

## Materials and methods

This cross-sectional observational study was conducted at the Punjab Institute of Cardiology, Lahore, over 18 months (January 2022 to June 2023) after obtaining approval from the Institutional Review Board (IRB) of Punjab Institute of Cardiology, Lahore (approval no. RTPGME-Research-200), and written informed consent from all patients or their legal guardians. 

A total of 349 patients presenting with acute AW-STEMI were enrolled through a non-probability consecutive sampling technique. Baseline data related to demographics, clinical details, comorbidities, and electrocardiogram (ECG) were collected. AW-STEMI was diagnosed based on ischemic chest pain lasting >20 minutes, ST-segment elevation in contiguous leads (≥2 mm in men, ≥1.5 mm in women in V1-V3), and elevated cardiac troponin levels (≥0.05 ng/mL). RBBB was defined as QRS duration >120 ms, rsR' pattern in V1, broad S waves in I, V5, V6, and ST depression/T-wave inversion in V1-V3. Patients aged ≥18 years diagnosed with AW-STEMI were included if they had ECG-confirmed STEMI with or without RBBB and were admitted within 12 hours of symptom onset. Exclusion criteria comprised prior STEMI, chronic arrhythmias (e.g., atrial fibrillation, permanent pacemaker dependency), recent cardiac surgery (within the last three months), pre-existing chronic RBBB, and major non-cardiac illnesses. Major non-cardiac illnesses were defined as advanced malignancy with a prognosis of <6 months, severe chronic kidney disease (CKD) requiring dialysis (stage 5 CKD), advanced liver disease with Child-Pugh Class C cirrhosis, and end-stage heart failure (NYHA Class IV) unrelated to acute ischemia. 

Patients were classified into two groups upon admission: Group 1 included those diagnosed with AW-STEMI with RBBB on the initial ECG, while Group 2 (control group) consisted of AW-STEMI patients without RBBB. The primary outcome was all-cause in-hospital mortality. Secondary outcomes included CHB, defined as sustained atrioventricular (AV) block requiring pacing; cardiac arrest, characterized by ventricular tachycardia/ventricular fibrillation (VT/VF) or pulseless electrical activity (PEA) requiring cardiopulmonary resuscitation (CPR); post-myocardial infarction (MI) angina, identified by recurrent chest pain with ischemic ECG changes or a rise in troponin levels; cardiogenic shock, assessed using the Society for Cardiovascular Angiography and Interventions (SCAI) criteria, defined by systolic blood pressure (SBP) <90 mmHg, lactate >2 mmol/L, and the requirement of vasopressors or intra-aortic balloon pump (IABP) support; arrhythmias, including atrial fibrillation (AF) and sustained VT/VF; and hospital stay duration, measured in days from admission to discharge or death.

Data analysis was conducted using IBM SPSS Statistics for Windows, Version 26.0 (released 2019, IBM Corp., Armonk, NY). Qualitative variables were presented as frequencies and percentages and compared using the Chi-square (χ²) test. Quantitative variables were expressed as mean ± standard deviation (SD) and analyzed using the independent t-test. Binary logistic regression analysis was performed to calculate adjusted odds ratios (OR) with 95% confidence intervals (CI), adjusting for potential confounders, including age, sex, hypertension, diabetes mellitus, dyslipidemia, prior myocardial infarction, and previous PCI or CABG. Confounding variables, including baseline characteristics, were stratified, and a post-stratification Chi-square test was applied to assess their impact on mortality and other outcomes. A p-value of less than 0.05 was considered statistically significant.

## Results

Patients with STEMI and RBBB had a comparable mean age between groups, with no significant differences in hypertension, diabetes, CKD, dyslipidemia, smoking status, prior myocardial infarction, or prior PCI/CABG. Peak troponin-I levels were significantly higher in the RBBB group, while LVEF was significantly lower. BMI was also higher in RBBB patients. Primary PCI was more frequent in non-RBBB patients, although the difference was not significant. Thrombolysis was more commonly performed in the RBBB group, while the use of medical management was similar between groups. The most common culprit artery was the LAD in both groups, followed by the LCx, with minimal involvement of the RCA and multivessel disease. Detailed results are shown in Table 1.

**Table 1:** Baseline characteristics of patients with STEMI based on the RBBB status. STEMI: ST-elevation myocardial infarction, RBBB: right bundle branch block, BP: blood pressure, LVEF: left ventricular ejection fraction, PCI: percutaneous coronary intervention, CABG: coronary artery bypass grafting, LAD: left anterior descending artery, RCA: right coronary artery, LCx: left circumflex artery. Continuous variables (age, BMI, symptoms to door time, systolic BP, diastolic BP, peak troponin-I, LVEF) were analyzed using independent t-tests, with results presented as t(df), p-value, and 95% confidence interval (CI). Categorical variables (hypertension, diabetes mellitus, chronic kidney disease, dyslipidemia, smoking status, prior myocardial infarction, prior PCI or CABG, culprit artery, reperfusion strategy) were analyzed using the Chi-square (χ²) test, with df and p-values reported. 95% CI is provided only for continuous variables. A p-value ≤ 0.05 was considered statistically significant.

Variables	STEMI with RBBB (n = 50)	STEMI without RBBB (n = 299)	t or χ² (df), p-value; 95% CI (where applicable)
Age (years)	52.34 ± 14.90	50.10 ± 13.16	t (347) = 1.090, p = 0.276; (-1.798, 6.270)
BMI (kg/m²)	28.62 ± 3.42	27.13 ± 3.73	t (347) = 2.279, p = 0.008; (0.383, 2.599)
Symptoms to door time (hours)	4.48 ± 2.10	4.59 ± 2.03	t (347) = -0.364, p = 0.716; (-0.7271, 0.4998)
Systolic BP at presentation (mmHg)	106.00 ± 21.85	106.89 ± 21.25	t (347) = -0.273, p = 0.785; (-7.302, 5.523)
Diastolic BP at presentation (mmHg)	77.60 ± 13.33	77.39 ± 12.79	t (347) = 0.106, p = 0.916; (-3.659, 4.076)
Peak troponin I (ng/mL)	33.54 ± 6.21	24.35 ± 9.03	t (347) = 6.925, p < 0.001; (6.581, 11.803)
LVEF (%)	40.92 ± 5.35	45.75 ± 5.97	t (347) = -5.373, p < 0.001; (-6.605, -3.065)
Hypertension, n (%)	24 (48.0%)	123 (41.1%)	χ² = 0.828, df = 1, p = 0.363
Diabetes mellitus, n (%)	13 (26.0%)	81 (27.1%)	χ² = 0.026, df = 1, p = 0.872
Chronic kidney disease, n (%)	9 (18.0%)	74 (24.7%)	χ² = 1.076, df = 1, p = 0.299
Dyslipidemia, n (%)	22 (44.0%)	116 (38.8%)	χ² = 0.485, df = 1, p = 0.486
Smoking status, n (%)	15 (30.0%)	83 (27.8%)	χ² = 0.419, df = 1, p = 0.517
Prior myocardial infarction, n (%)	5 (10.0%)	22 (7.4%)	χ² = 0.096, df = 1, p = 0.757
Prior PCI or CABG, n (%)	4 (8.0%)	28 (9.4%)	χ² = 0.096, df = 1, p = 0.757
Culprit artery (LAD), n (%)	44 (88.0%)	245 (81.9%)	χ² = 1.105, df = 3, p = 0.776
Culprit artery (LCx) n (%)	4 (8.0%)	36 (12.0%)	
Culprit artery (RCA) n (%)	1 (2.0%)	9 (3.0%)	
Culprit artery (multivessel) n (%)	1 (2.0%)	9 (3.0%)	
Reperfusion strategy (primary PCI), n (%)	27 (54.0%)	196 (65.6%)	χ² = 4.220, df = 2, p = 0.121
Reperfusion strategy (thrombolysis) n (%)	15 (30.0%)	53 (17.7%)	
Reperfusion strategy (medical management) n (%)	8 (16.0%)	50 (16.7%)	

Mortality was notably higher in the RBBB group (16% vs. 5.7%, p = 0.009, OR = 3.160, 95% CI: 1.284-7.777). Similarly, cardiogenic shock occurred more frequently in these patients (36% vs. 16.4%, p = 0.003, OR = 2.672, 95% CI: 1.394-5.120). Arrhythmias, including atrial fibrillation and supraventricular tachycardia, were significantly more common among RBBB patients (42% vs. 19.7%, p = 0.001). Cardiac arrest and CHB were observed at higher rates in RBBB patients, although these differences did not reach statistical significance. Post-MI angina was also more frequent in RBBB patients (Table 2).

**Table 2:** Comparison of outcomes of patients with STEMI based on the RBBB status. STEMI: ST-elevation myocardial infarction, RBBB: right bundle branch block, OR: odds ratio, CI: confidence interval, AF: atrial fibrillation, SVT: supraventricular tachycardia. The Chi-square (χ²) test was used to compare categorical outcomes between the RBBB and non-RBBB groups, with degrees of freedom (df) and corresponding p-values reported. Adjusted odds ratios (OR) with 95% confidence intervals (CI) were calculated using binary logistic regression analysis. Adjustments were made for potential confounders, including age, sex, hypertension, diabetes mellitus, dyslipidemia, prior myocardial infarction, and previous PCI or CABG.

Outcomes	RBBB group (n = 50), n (%)	Non-RBBB group (n = 299), n (%)	Chi-square (χ²), df, p-value	Adjusted OR (95% CI)
In-hospital mortality	8 (16%)	17 (5.7%)	χ² = 6.853, df = 1, p = 0.009	3.160 (1.284–7.777)
Complete heart block	6 (12%)	24 (8%)	χ² = 0.861, df = 1, p = 0.354	1.562 (0.605–4.038)
Cardiac arrest	8 (16%)	34 (11.4%)	χ² = 0.867, df = 1, p = 0.354	1.485 (0.643–3.426)
Post-MI angina	12 (24%)	46 (15.4%)	χ² = 2.295, df = 1, p = 0.170	1.631 (0.811–3.278)
Cardiogenic shock	18 (36%)	49 (16.4%)	χ² = 10.622, df = 1, p = 0.003	2.672 (1.394–5.120)
Arrhythmias (AF, SVT, etc.)	21 (42%)	59 (19.7%)	χ² = 16.919, df = 2, p = 0.001	2.946 (1.569–5.529)

Post-stratification analysis of confounding variables on mortality revealed no significant impact of hypertension, with three (30.0%) out of 10 hypertensive STEMI patients with RBBB versus seven (15.3%) out of 137 without RBBB succumbing (p = 0.226). Similarly, diabetes mellitus did not significantly affect mortality, as three (20.0%) out of 15 diabetic STEMI patients with RBBB versus 12 (12.7%) out of 79 without RBBB died (p = 0.450). Conversely, CKD was strongly associated with mortality, with all three (100.0%) STEMI patients with RBBB versus six (7.5%) out of 80 without RBBB dying (p < 0.001). Among non-CKD patients, mortality rates were 5 (22.7%) out of 22 with RBBB versus 17 (14.8%) out of 244 without RBBB, although not statistically significant (p = 0.321). Prior myocardial infarction did not significantly influence mortality (p = 0.238), but among patients without a history of infarction, mortality was significantly higher in the RBBB group (8 (40.0%) out of 20 versus 12 (12.3%) out of 302, p = 0.001). Prior PCI or CABG had no significant effect, with 0 (0.0%) out of four with RBBB versus five (14.8%) out of 27 without RBBB dying (p = 0.358). Reperfusion strategy was a key determinant of mortality. Primary PCI showed no significant difference, with one (5.6%) out of 18 with RBBB versus 17 (94.4%) out of 205 without RBBB dying (p = 0.374). However, thrombolysis resulted in markedly higher mortality in the RBBB group, with all four (100.0%) STEMI patients versus 0 (0.0%) out of 64 without RBBB dying (p < 0.001). Similarly, medical management led to 100.0% mortality (3/3) in RBBB versus 0 (0.0%) out of 55 in non-RBBB patients (p < 0.001).

## Discussion

The prevalence of RBBB in acute AW-STEMI has been extensively studied, with varying frequencies. The current study reported RBBB in 17.4% of patients with anterior STEMI. Contrarily, Shrivastav et al. (2021), utilizing the National Inpatient Sample (NIS) database, identified RBBB in 1.8% of AW-STEMI cases among 1,075,875 hospital admissions [5]. In addition, some regional studies found a higher prevalence of RBBB in STEMI (21.3-35.5%) than in the present study [10,11].

The mean age of patients with RBBB in the present study was 52.34 ± 14.90 years, compared to 50.10 ± 13.16 years in the non-RBBB group (p = 0.451), indicating no significant difference. These findings align with those of Kamran et al. (2023), who reported a similar mean age (47.51 ± 10.56 years in RBBB vs. 49.99 ± 12.82 years in non-RBBB patients) [11]. However, Shrivastav et al. (2021) and Figueroa-Triana et al. (2021) observed an older cohort, with mean ages of 68.5 years and 66 years, respectively [4,5].

Comorbidities such as hypertension, diabetes mellitus, and CKD were prevalent in both groups but did not show statistically significant differences. In the present study, hypertension was reported in 48.0% of RBBB patients versus 41.1% of non-RBBB patients (p = 0.363), aligning the results of Kamran et al. (2023) (51.3% vs. 50.0%) [11]. Similarly, DM was observed in 26.0% of RBBB patients vs. 27.1% in non-RBBB patients (p = 0.872), consistent with Kamran et al. (2023) (48.8% vs. 51.3%) and Figueroa-Triana et al. (2021) (25.9% vs. 24.0%) [4,11]. These findings indicate that although these comorbidities are common in STEMI patients, they do not independently increase the risk to RBBB. The present study revealed significantly higher peak troponin-I levels in STEMI patients with RBBB compared to those without RBBB (p = 0.012, 95% CI: 6.581-11.803). These results were consistent with previous studies [11]. In Zameer et al. (2022), LVEF was lower in the RBBB group (p = 0.407), although it was not statistically significant [10]. By contrast, Sarker et al. (2018) (38.21 ± 5.14% vs. 40.24 ± 4.69%, p < 0.05) and Brilakis et al. (2001) (38 ± 16% vs. 50 ± 15%, p < 0.0001) found significantly lower LVEF in RBBB patients [10,12,13].

This study reported in-hospital mortality rate of 16% in RBBB patients versus 5.7% in non-RBBB patients (p = 0.009, OR = 3.160, 95% CI: 1.284-7.777), supported by the results from previous studies where Sarker et al. (2018) who observed a similar trend (21.9% vs. 7.9%, p < 0.05) [13]. Kamran et al. (2023) also documented 21.3% mortality in RBBB patients compared to 6.3% in non-RBBB patients (p = 0.006) [11]. Brilakis et al. (2001) reported 13.3% mortality in RBBB patients, lower than our findings but still indicating increased risk compared to patients without BBB (9.1% mortality, p = 0.11) [12]. Juárez et al. (2010) reported a 20% mortality rate in STEMI patients with RBBB (OR = 1.70, 95% CI: 1.19-2.42, p < 0.003), while Shrivastav et al. (2021) observed a higher mortality in AW-STEMI patients with RBBB (15.3% vs. 9.2%), Xiang et al. (2016) found RBBB significantly increased in-hospital mortality (OR = 1.94, 95% CI: 1.60-2.37, p = 0.002), and Alkindi et al. (2020) reported a markedly higher all-cause mortality in RBBB patients (p = 0.001, adjusted OR = 5.14, 95% CI: 3.90-6.70) [5,14-16]. Collectively, these studies reported that RBBB in STEMI is associated with higher mortality, likely due to larger infarct size, greater myocardial dysfunction, conduction abnormalities, and higher rates of cardiogenic shock and arrhythmias, necessitating early identification, aggressive hemodynamic support, and optimized reperfusion strategies.

Cardiogenic shock remains significantly more prevalent in STEMI patients with RBBB, indicating severe myocardial dysfunction and hemodynamic instability. The present study observed a 36% incidence in RBBB patients compared to 16.4% in non-RBBB patients (p = 0.003, OR = 2.672, 95% CI: 1.394-5.120), reinforcing the association between RBBB and circulatory failure. These results are comparable with Sarker et al. (2018) (31.3% vs. 13.2%) and Alkindi et al. (2020) (10.6% vs. 1.7%) [13,15]. In addition, Shaik et al. (2023) reported a markedly higher rate (54.5% vs. 6.4%), suggesting more extensive infarction and left ventricular dysfunction [17].

Post-MI angina was more prevalent in RBBB patients in the present study (24% vs. 15.4%, p = 0.170, OR = 1.631, 95% CI: 0.811-3.278), although the difference did not reach statistical significance. This contrasts with previous studies that reported a strong association between RBBB and post-MI angina. Sarker et al. (2018) found a significantly higher incidence (53.1%, p = 0.002), indicating greater residual ischemia and infarct size in their cohort [13]. Xiang et al. (2016) reinforced this in a meta-analysis, showing increased post-MI ischemia and reinfarction risk in RBBB patients. Juárez et al. (2010) demonstrated that RBBB patients had persistent ST-segment depression (OR = 3.43, 95% CI: 1.51-7.78, p = 0.003), suggesting inadequate myocardial recovery [14]. Zameer et al.'s (2022) study found a higher incidence of post-MI angina in AW-STEMI with RBBB, particularly in those with multivessel disease [10].

Patients with STEMI and RBBB had a higher frequency of arrhythmias, which highlights the increased risk of electrical instability. Arrhythmias occurred in 42% of RBBB patients compared to 19.7% in non-RBBB patients (p = 0.001, OR = 2.946, 95% CI: 1.569-5.529) in the current study. These results are supported by Juárez et al. (2010), who identified conduction abnormalities as independent predictors of mortality in RBBB patients [14]. Xiang et al. (2016) further confirmed this relation, reporting a significantly increased risk of arrhythmias in AMI patients with RBBB. Similarly, Shaik et al. (2023) reported VT in 27.2% of RBBB patients compared to only 2.6% in non-RBBB patients, emphasizing the vulnerability of this subgroup to ventricular arrhythmias [17]. Brilakis et al. (2001) and Alkindi et al. (2020) also observed a significantly higher incidence of VT and VF in RBBB patients, supporting the association between conduction defects and adverse cardiac events [12,15].

The incidence of cardiac arrest in RBBB patients was higher (16% vs. 11.4%, p = 0.354, OR = 1.485, 95% CI: 0.643-3.426), although statistical significance was not achieved. Despite this, the trend holds clinical relevance, as cardiac arrest remains a critical complication with severe prognostic implications in STEMI patients. This contrasts with Shaik et al. (2023), who reported a markedly higher VT incidence in qRBBB patients (27.2% vs. 2.6%), and Brilakis et al. (2001), linking RBBB to increased VT/VF episodes [12,17]. Xiang et al. (2016) identified RBBB as a risk factor for in-hospital mortality due to fatal arrhythmias [16]. Juárez et al. (2010) found higher AV block rates (OR = 2.99, 95% CI: 1.9-3.1), while Alkindi et al. (2020) reported increased ventricular tachyarrhythmias (7.3% vs. 2.2%, p = 0.001) [14,15]. Shrivastav et al. (2021) associated RBBB with higher acute heart failure (21.1%) and cardiogenic shock (15.6%) rates, both major contributors to cardiac arrest [5]. Zameer et al. (2022) linked increased ST-segment elevation (p = 0.083) and lower ejection fraction (p = 0.032) with greater arrhythmic risk, reinforcing the need for vigilant cardiac monitoring in RBBB patients [10].

This study found that CHB was more common in RBBB patients (12%) compared to (8%) in non-RBBB patients (p = 0.345, OR = 1.562, 95% CI: 0.605-4.038), but the difference is not statistically significant. This contradicts with Juárez et al. (2010), who found that third-degree AV block as significantly more common in RBBB patients (OR = 2.99, 95% CI: 1.9-3.1) [14]. Similarly, Alkindi et al. (2020) found a higher rate of conduction abnormalities requiring pacemaker implantation in RBBB patients [15]. Xiang et al. (2016) and Lorenzová and Widimský (2009) confirmed RBBB’s association with AV block and poor prognosis [16,18]. These findings support the need for continuous monitoring and early pacemaker consideration in STEMI patients with RBBB.

CKD was strongly associated with increased in-hospital mortality (p < 0.001), whereas other comorbidities, including hypertension and diabetes, did not show a significant impact. Contrarily, previous studies reported that diabetes and hypertension contributed to worse outcomes [5]. Brilakis et al. (2001) reported that RBBB patients often had pre-existing cardiovascular disease, higher Killip class, and lower ejection fraction [12]. Similarly, Zameer et al. (2022) demonstrated that higher Killip class (p < 0.001), multi-vessel disease (p = 0.014), and decreased ejection fraction (p = 0.032) were associated with worse clinical outcomes in patients with RBBB [10]. Reperfusion strategies also affected prognosis, with PCI decreasing mortality rate (12.5% vs. 100%, p < 0.001), consistent with previous studies reporting that suboptimal use of PCI in RBBB patients may contribute to higher mortality [10,12,15]. Kamran et al. (2023) found that RBBB patients undergoing thrombolysis had significantly higher mortality, reinforcing PCI’s beneficial role [11]. The variation in findings could be attributed to differences in study populations, variations in diagnostic criteria, or regional disparities in healthcare access and treatment strategies. In addition, differences in the severity and duration of comorbidities such as diabetes and hypertension may have influenced outcomes across studies. The management of CKD in patients with RBBB requires careful optimization of fluid balance, electrolyte homeostasis, and early risk stratification. Individualized revascularization approaches and close monitoring for arrhythmias are crucial in improving outcomes.

This study provides useful insights into the prognostic impact of RBBB in AW-STEMI. However, limitations include a single-center setting, which may bound generalizability, and a relatively small sample size. Future research should focus on longitudinal multicenter studies considering long-term follow-up. In addition, exploring the role of advanced therapies, such as early electrophysiological interventions, could further optimize management strategies for STEMI patients with RBBB.

## Conclusions

AW-STEMI with RBBB was associated with higher in-hospital mortality, cardiogenic shock, and arrhythmias, reflecting greater hemodynamic instability. Although cardiac arrest, post-MI angina, and complete heart block were more frequent, their clinical relevance remains despite lacking statistical significance. CKD strongly predicted mortality, consistent with prior research linking it to ischemic injury and arrhythmias. Primary PCI significantly reduced mortality, aligning with previous findings, while thrombolysis showed worse outcomes, emphasizing the need for timely PCI and individualized reperfusion strategies in this high-risk group.
